# Uptake and reduction of Se(IV) in two heterotrophic aerobic *Pseudomonads* strains isolated from boreal bog environment

**DOI:** 10.3934/microbiol.2017.4.798

**Published:** 2017-10-10

**Authors:** Merja Lusa, Jenna Knuutinen, Malin Bomberg

**Affiliations:** 1 Department of Chemistry, Radiochemistry, University of Helsinki, P.O. Box 55, 00014 Helsinki, Finland; 2 VTT Technical Research Centre of Finland, Espoo, Finland

**Keywords:** environmental, metabolic processes, *Pseudomonads*, soil, ecology, selenium, selenite, selenite, boreal bog

## Abstract

Selenite (Se(IV), SeO_3_^2−^) uptake and the effect of selenite supplement on protein synthesis was investigated in two *Pseudomonas* sp. strains isolated from a boreal bog. These aerobic bacteria efficiently reduced Se(IV) with intracellular reduced Se^0^ observed in the cytoplasm under dark aerobic conditions. The proteome analysis of Se(IV) supplement and temperature responses by SDS-PAGE gel electrophoresis showed variations in the protein expression on the 40–60 kDa regions following these stress factors, probably through enzymes associated to oxidative stress or temperature adaptation. NO_3_^−^/NO_2_^−^/SO_4_^2−^ addition enhanced Se(IV) uptake in both bacteria, but Se(IV) uptake sustained also under sulphur and nitrogen starvation. Our findings suggest two different transport mechanisms for Se(IV) uptake in these *Pseudomonas* sp. strains; a low affinity transport system up-regulated by NO_3_^−^/NO_2_^−^/SO_4_^2−^ and a distinct Se(IV)O_3_^2−^ regulated transport system. Following transport, Se(IV) is reduced in the cytoplasm, forming Se^0^ granules, visible in TEM and verified using EDX.

## 1. Introduction

^79^Se is one of the high priority radionuclides in the long-term biosphere safety assessment of spent nuclear fuel [1]. In addition, considerable amounts of stable selenium enter the environment via anthropogenic activities including coal combustion, mining, refining of sour crude oils and agricultural irrigation of seleniferous soils [2]–[5]. The biological effects of selenium make it a particular hazard for environmental releases and both its environmental mobility and biological effects are mainly controlled by its chemical speciation [6],[7],[8]. Although selenium is an essential micronutrient for animals and humans, the higher valence states (selenate, Se(VI)O_4_^2−^ and selenite, Se(IV)O_3_^2−^) are toxic at elevated concentrations [9],[10] The toxic character of these oxyanionic compounds is related to their oxidant capacity [11]. Selenite is a highly reactive species of selenium and reacts in particular with thiol groups found in glutathione (GSH). This explains, at least in part, its toxic character [11]. Glutathione and selenite spontaneously react to produce several selenium-containing compounds, including selenodiglutathione, glutathioselenol, hydrogen selenide and elemental selenium [12]. This reaction produces also highly toxic oxygen species like H_2_O_2_ and O^2−^ [13]. The reduction of the soluble oxyanions selenate and selenite converts selenium into the less toxic, insoluble Se^0^. In the environment, selenate and selenite reduction is mainly mediated by microorganisms [14],[15],[16], although slow abiotic reduction of selenium oxyanions can also occur under highly reducing conditions in the presence of Fe(II,III) oxides [17]. Some anaerobic and phototrophic aerobic selenium respiring bacteria have previously shown to be able to use selenium oxyanions as terminal electron acceptors precipitating insoluble elemental Se^0^ particles [11],[18]–[23]. Following reduction, both intracellular and extracellular selenium granules have been found in phylogenetically and physiologically distinct bacteria like *Chromatium vinosum*, *Desulfovibrio desulfuricans*, *Sulfospirillum barnesii*, *Bacillus selenitireducens*, *Selenihalanaerobacter shriftii*, *Shewanella oneidensis* MR-1, *Paenibacillus selenitireducens* sp. nov., and *Cupriavidus metallidurans* CH34 (formerly *Ralstonia metallidurans* CH34) [22],[24],[25]. Microbial reduction of selenium oxyanions produces red elemental selenium with crystalline or amorphous structures and the elemental Se^0^ particles formed by Se-respiring *Sulfurospirillum barnesii*, *Bacillus selenitireducens* and *Selenihalanaerobacter shriftii* have been shown to be structurally distinctive from elemental selenium formed by chemical synthesis [22],[26]. In addition there are indications that these different species of Se-reducing bacteria produce Se^0^ biominerals with different atomic structures [22]. However, while Se-reduction has been shown to be an environmentally important process in diverse terrestrial and aquatic environments, the mechanisms of Se^0^ biomineralization are poorly understood and the molecular factors behind Se reduction reactions have not been identified [27].

Previously, we found the two heterotrophic aerobic *Pseudomonas* strains, used in this study, to remove ^75^Se(IV)O_3_^2−^ from solutions under different nutrient conditions [28]. In this study, the factors affecting the uptake and reduction of Se oxyanions by these two *Pseudomonas* strains, PS-0-L and T5-6-I isolated from a boreal bog environment [29], was examined using batch experiments, transmission electron microscopy (TEM) and energy dispersive X-ray spectroscopy (EDX). In addition, SDS-PAGE was used to study the proteins expressed in these bacteria in the presence of both selenium oxyanions Se(IV)/Se(VI) as well as other anionic macronutrients (sulphate (SO_4_^2−^), sulphite (SO_3_^2−^), nitrate (NO_3_^−^), nitrite (NO_2_^−^)).

## 2. Materials and Method

### 2.1. Bacterial isolates

**Figure 1.**
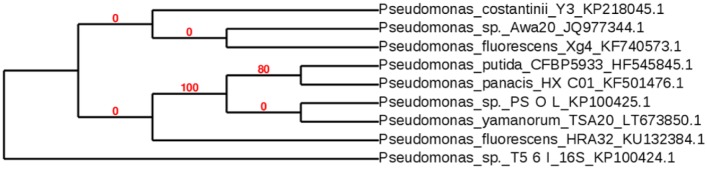
Phylogenetic tree constructed from the 16S rRNA gene sequences of Pseudomonas PS-0-L and T5-6-I using the PhyML tool and the Hky85 substitution model. The topology of the tree was tested using aLRT [30],[31].

The two *Pseudomonas* sp. strains PS-0-L and T5-6-I (Figure 1) used in this study were isolated from the peat of Lastensuo Bog in June 2013 and identified using 16S rRNA gene sequencing as described in Lusa et al. [29]. Both rod shaped oxidase-negative and catalase-positive strains belonged to the genus *Pseudomonas* and stained Gram negative. The utilization of substrates by these two strains was tested using the RapID™ ONE and RapID™ NF Plus systems (Remell) [29] and both strains T5-6-I and PS-0-L had similar phenotypic biochemical characteristics, with the exception of the capability to hydrolyse acrylamide; while T5-6-I could hydrolyse γ-glutamyl β-naphthylamide and Pyrrolidine-β-naphthylamide, PS-0-L could not. Both strains were able to reduce nitrate and hydrolyze arginine, fatty acids and acrylamids. The sequences have been deposited in Genbank under accession numbers KP100424 and KP100425.

### 2.2. Bacterial culture conditions and preparation ^75^[Se]-selenite uptake assays

Isolated bacterial strains were cultured aerobically on sterile PCA growth plates (PCA, Merckoplate®) at 20 °C in the dark and the strains were recultivated on new plates weekly. A batch method utilizing both radioactive tracer and stable selenium salts was used in following experiments. In radioactive tracer experiments Na_2_^75^Se(IV)O_3_ with 2.7 × 10^−8^ M stable Se(IV) carrier was used. In non-radioactive experiments Na_2_Se(IV)O_3_ and Na_2_Se(VI)O_4_ salts were used.

To study the transport systems present in these bacteria different nutrient and incubation conditions were used. As active transport systems are typically affected by glucose and various inhibitors, we tested the effect of glucose and L-cysteine on Se(IV) uptake using medium A (0.5% peptone + 0.25% yeast extract + 4 µM phosphate buffer) to which 0.1% glucose was added or medium B (1% Yeast extract + 4 µM phosphate buffer) to which 100–5000 µM L-cysteine was added. Control tests were also prepared with both media (A and B) without the addition of glucose or L-cysteine. The cell growth after L-cysteine addition was monitored by measuring cell density at 660 nm. As the used strains reach mid and late stationary phase after 4 to 7 days incubation, cell density was measured at t = 0 and after 7 days of incubation. The possible toxic effects of Se(IV) were tested by increasing Se(IV) carrier concentration in the 1% Tryptone medium from 0.3 mM to 60 mM (medium C, in 4 µM phosphate buffer). The effect of Se(IV) carrier concentration on the following ^75^Se(IV) uptake was monitored after 24 h of incubation. pH influence on Se(IV) uptake by *Pseudomonas* sp. strain PS-0-L was in addition tested by adjusting the pH of 1% Yeast extract solution to between 4 and 7.2, which represents the naturally occurring pH in the boreal bog environment, with 1 M HCl/1 M NaOH (medium D).

Bacterial colonies from the PCA plates were moved into sterile 0.01 M phosphate buffer using a sterile loop and bacterial mass was added until the turbidity of the solution corresponded to a McFarland standard No.6, which corresponds to an approximate cell density of 18 × 10^8^ Cells/mL. The suspensions were weighted and 2 ml of this suspension was added to 5 ml of medium A–C, after which 200 Bq of ^75^[Se(IV)O_3_^2−^]-label per suspension was added. All tests were conducted using triplicate samples. The suspensions were incubated at 4 °C, 20 °C or 37 °C depending on the set of experiments in the dark for 7 days, after which the suspensions were filtered through a 0.2 µm sterile membrane filter and the activity of the resulting solutions was measured using a NaI(Tl)-gammaspectrometer (Wizard® automatic gammacounter, PerkinElmer). In addition, suspensions without added bacteria were prepared accordingly and measured to assure that no sorption or precipitation of ^75^Se(IV) occurred on laboratory equipment, filters or nutrient broth solutions. The uptake of ^75^Se(IV) induced by the bacterial cells was calculated from the difference between initial and final (after filtration) Se(IV) concentration in the solution and expressed as correlation coefficient (L kg^−1^ DW):

$$\left[K_{d}=\frac{A_{i}-A_{f}}{A_{f}}\times \frac{V}{m}\right]$$ (1)

where A_i_ (Bq L^−1^) and A_f_ (Bq L^−1^) are the initial activity and final activity concentration of the solution, V (L) is the solution volume, and m (kg DW) is the sample mass at t = 0. All calculations were performed using dry mass determined at 105 °C and the uncertainty of the measurements was expressed as standard deviation of three parallel determinations.

### 2.3. Induction of protein synthesis

The role of macronutrients on selenite uptake was examined using solutions E1–E14 (Table 1), in which 3–6 µM of SeO_3_^2−^, SeO_4_^2−^, NO_3_^−^, NO_2_^−^, SO_4_^2−^ and/or SO_3_^2−^ were added in 1% Tryptone solution, pH 7 in phosphate buffer, depending on the test series. E-solutions were used both in the tracer experiments with ^75^Se(IV) as well as in the protein induction and TEM experiments using stable NaSe(IV)O_3_ or NaSe(VI)O_4_ as described below.

The stress responses generated by Se(IV) or Se(VI)O_4_^2−^ addition were examined to identify potentially important proteins involved in Se(IV) metabolism and reduction. Bacterial cultures under aerobic conditions at 4 °C or 20 °C in the dark were treated with 0–6 mM Se(IV) or 0–6 mM Se(VI) and 0–3 mM NO_2_^−^/NO_3_^−^/SO_3_^2−^/SO_4_^2−^ (media E1–E14, Table 1). The two *Pseudomonas* sp. strains used in our study are relatively slowly growing boreal strains and according to the growth curves they reach mid-exponential growth phase after 1–2 days incubation and stationary growth phase at 4–7 days (data not shown). Therefore incubation times of 24 h (exponential phase) and 7 days (late stationary phase) were used in the experiments. After the incubation period, a 100 µl aliquot sample was used for dilution plating on PCA plates to ensure the viability of the bacterial cells after Se(IV) treatment. The soluble protein fractions from control untreated, SeO_3_^2−^/SeO_4_^2−^/NO_2_^−^/NO_3_^−^/SO_3_^2−^/SO_4_^2−^ treated and SeO_3_^2−^/SeO_4_^2−^ + NO_2_^−^/NO_3_^−^/SO_3_^2−^/SO_4_^2−^ treated cells were isolated using the BugBuster™ method according to the manufacturer's instructions and isolated proteins were separated with SDS-PAGE using pre-cast ClearPage™ SDS 12% gels with constant voltage of 175 V for 45 minutes. SDS-PAGE samples were prepared following the manufacturer's instructions for ClearPage™ gels, using 1 µL of 10× DTT per 10 µL of sample volume. TEO-Tricine (ClearPAGE SDS Running Buffer) was used as running buffer. The proteins were visualized using the Blue BANDIT™ protein stain.

**Table 1.** Media E1–E14; Se(IV) and Se(VI) and macronutrient solutions used in the tracer and protein induction experiments. The micro- and macronutrients were added into 1% Tryptone solution.

Macronutrient solutions		Selenite solutions		Selenate solutions	
E1	6 mM NO_2_^−^	E5	6 mM SeO_3_^2−^	E10	6 mM SeO_4_^2−^
E2	6 mM NO_3_^−^	E6	3 mM SeO_3_^2−^ + 3 mM NO_2_^−^	E11	3 mM SeO_4_^2−^ + 3 mM NO_2_^−^
E3	6 mM SO_3_^2−^	E7	3 mM SeO_3_^2−^ + 3 mM NO_3_^−^	E12	3 mM SeO_4_^2−^ + 3 mM NO_3_^−^
E4	6 mM SO_4_^2−^	E8	3 mM SeO_3_^2−^ + 3 mM SO_3_^2−^	E13	3 mM SeO_4_^2−^ + 3 mM SO_3_^2−^
		E9	3 mM SeO_3_^2−^ + 3 mM SO_4_^2−^	E14	3 mM SeO_4_^2−^ + 3 mM SO_4_^2−^

### 2.4. TEM and EDX analyses

TEM (Transmission Electron Microscopy) and EDX (Energy Dispersive X-Ray Spectroscopy) (Electron Microscopy Unit, Institute of Biotechnology, University of Helsinki) were used to identify intra- and extracellular Se^0^ granules in bacterial cells grown with 6–60 mM Se(IV) or Se(VI)O_4_^2−^. Cells were grown at 20 °C and an incubation time of seven days was used. After 7 days incubation an 1 mL subsample of the growing suspension was taken and the cells were fixed in 1 mL of 5% glutaraldehyde. Cells were dehydrated through an ethanol series and then embedded in Taab hard epon and polymerized at 60 °C. Thin sections were cut using Leica ultracut UCT ultramicrotome (Leica Mikrosysteme GmbH, Austria) and collected on single-slot copper grids. Section thickness of 60 nm was used for the morphological examinations and 90 nm for elemental analysis. After double staining with uranyl acetate and lead citrate, the sections were examined using FEI Tecnai F20 at 200 kV under standard operating conditions. EDX was performed for unstained samples at 120 kV using a (live) counting time of 20 s.

## 3. Results

### 3.1. Effect of glucose, temperature and metabolic inhibitors on ^75^[Se]-selenite uptake

**Figure 2.**
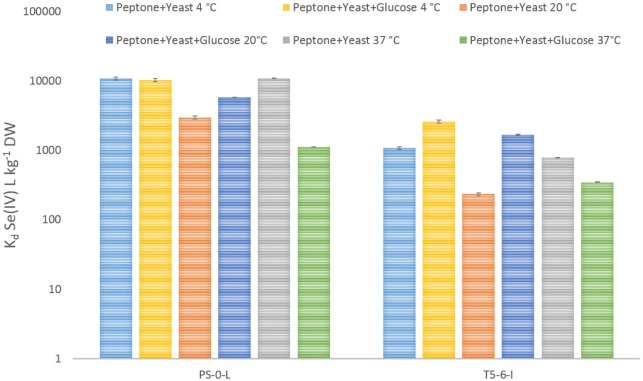
The effect of electron donor and temperature (4 °C, 20 °C and 37 °C) on Se(IV) uptake in the two *Pseudomonas* sp. strains PS-0-L and T5-6-I. The error indicated is a standard deviation of three parallel determinations.

We tested the effect of glucose (medium A) and L-cysteine (medium B) on the uptake of SeO_3_^2−^ by two strains of *Pseudomonas*, strains PS-0-L and T5-6-I previously isolated from an oligotrophic boreal fen [29]. Without glucose, the average Se(IV) removal from the solution containing peptone and yeast extract was 4400 L kg^−1^ by the two *Pseudomonas* strains. The addition of 0.1% glucose substantially increased the Se(IV)uptake in both *Pseudomonas* strains at 20 °C, showing 2-fold and 7-fold higher uptake rates by strains PS-0-L and T5-6-I, respectively (Figure 2). However, as we decreased the incubation temperature to 4 °C, a slight decrease of 4% was observed in Se(IV) uptake by strain PS-0-L after glucose addition. At elevated temperature of 37 °C, glucose addition decreased selenite uptake by both strains even more with an average decrease of 70% in selenite uptake after glucose addition. Change in the solution pH between 4–7.2 had no effect on Se(IV) uptake (data not shown).

**Figure 3.**
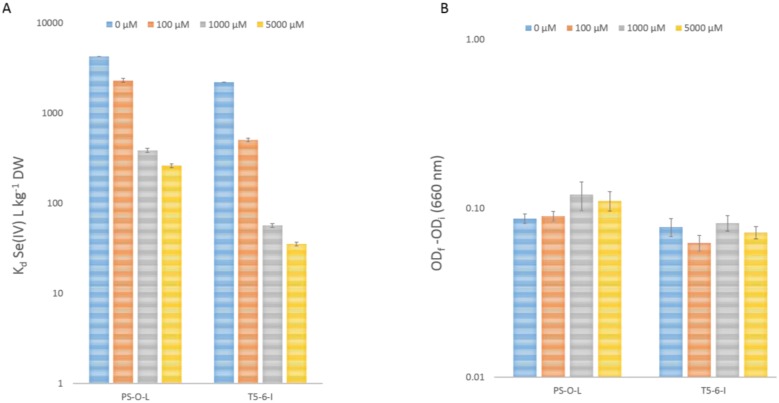
(A) The effect of 0–5000 µM L-cysteine addition (in 1% Yeast extract, temperature 20 °C) to Se(IV) utilization in *Pseudomonas* sp. strains PS-0-L and T5-6-I . (B) Cell growth of *Pseudomonas* sp. PS-0-L and T5-6-I (OD, 660 nm) after 0–5000 µM L-cysteine addition (in 1% Yeast extract, temperature 20 °C). The error indicated is a standard deviation of three parallel determinations.

If Se(IV) enters the cells by an active mechanism, it should also be affected by various metabolic inhibitors. We tested the effect of the sulphur-containing amino acid L-cysteine on the Se(IV) uptake. The addition of 100–5000 µM L-cysteine in 1% Yeast extract reduced Se(IV) uptake in both *Pseudomonas* sp. strains from an average of 3200 L/kg DW without L-cysteine to on average 35 L/kg DW after 5000 µM L-cysteine addition (Figure 3A). The cell growth after L-cysteine addition was monitored by measuring cell density at 660 nm at t = 0 and after 7 days incubation (Figure 3B) and impaired cell growth was not observed, compared to the cells grown without cysteine addition. Instead, for *Pseudomonas* PS-0-L a slightly elevated, on average 1.3 fold, cell growth rate was observed after 1000–5000 µM L-cysteine addition, compared to the cells grown in the absence of cysteine.

### 3.2. The effect of Se(IV) and Se(VI)O_4_^2−^ concentrations; TEM, EDX and ^75^[Se]-selenite uptake assays

**Figure 4.**
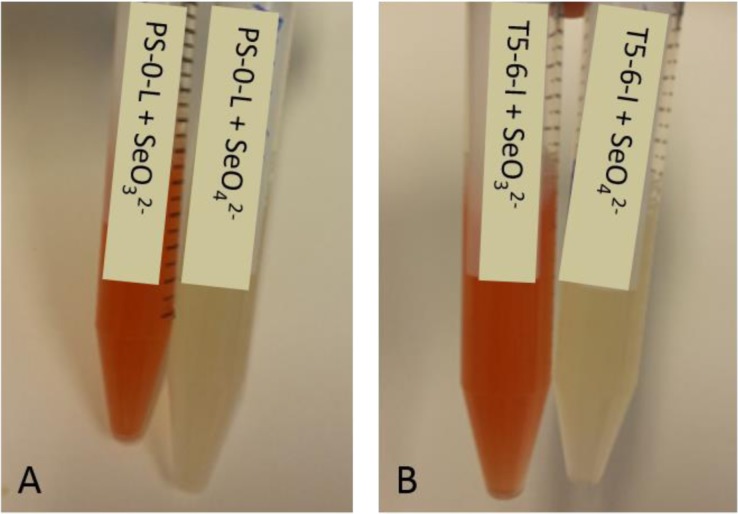
Formation of brick red reduced Se^0^ in Se(IV) containing broths incubated with (A) *Pseudomonas* sp. PS-0-L and (B) *Pseudomonas* sp. T5-6-I.

**Figure 5.**
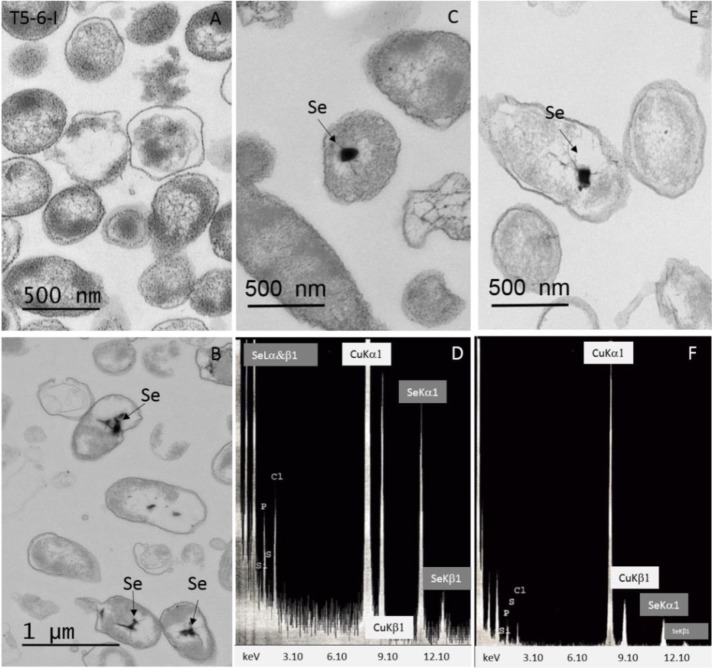
Thin-section micrographs of *Pseudomonas* sp. T5-6-I (A–D) and *Pseudomonas* sp. PS-0-L (E–F) grown under aerobic conditions in the dark in the absence (A) or in the presence of 6 mM Se(IV)O_3_^2−^ (B–F). Arrows show the presence of electron dense particles of reduced selenium (Se^0^). (D and F) EDX spectrum of electron-dense particles pointed out by arrows in B, C and E.

Both intra- and extracellular reduced Se^0^ granules have been found in distinct bacteria, but the generation process of these granules is still mainly unknown. To study the reduction hypothesis, we incubated both *Pseudomonas* sp. strains either with Se(IV) or Se(VI)O_4_^2−^. Both *Pseudomonas* sp. strains reduced Se(IV) to Se(0), as indicated by the appearance of a brick red precipitate after 7 days incubation in broths containing Se(IV) (Figure 4). For Se(VI), such precipitate was not visible to the eye (Figure 4). In the following TEM and EDX studies, intracellular Se^0^ granules were verified in both *Pseudomonas* sp. strains following Se(IV) reduction (Figure 5A, Figure 5B, Figure 5C, Figure 5E), with characteristic selenium peaks in X-ray spectrum (Figure 5D and 5F). However, the Se^0^ intensities proportional to the intracellular Se^0^ concentrations observed in EDX measurements were higher for the T5-6-I strain. In addition, for T5-6-I strain low intensities of intracellular Se^0^ was observed in the EDX measurements following incubation in 6 mM Se(VI) broth. However, in these cells metallic selenium was only barely detectable (data not shown). For PS-0-L similar structures after growing in Se(VI) containing broths were not observed.

**Figure 6.**
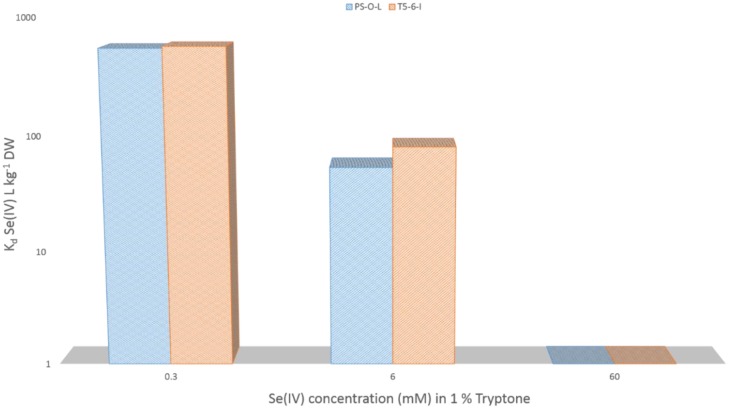
^75^Se(IV)O_3_^2−^ uptake in *Pseudomonas* sp. PS-0-L and T5-6-I grown under aerobic conditions in the dark in the presence of 0.3–60 mM Se(IV) in 1% Tryptone (t = 37 °C).

The sensitivity of these bacteria to Se(IV) was tested, by increasing the Se(IV) carrier concentration in the nutrient solution. As Se(IV) concentration was increased from 0.3 mM to 6 mM, the uptake of ^75^Se(IV) dropped to approximately one tenth from an average of 550 L kg^−1^ DW to 70 L kg^−1^ DW in both *Pseudomonas* sp. strains (Figure 6). 60 mM Se(IV) addition inhibited ^75^Se(IV) uptake completely. In addition we found changes in the cell morphology in the corresponding TEM images, in which the amount of dividing cells was clearly decreased and a vast majority of the cells were ruptured (data not shown).

### 3.3. The effect of anionic competitors on ^75^Se(IV) uptake and gene expression

**Figure 7.**
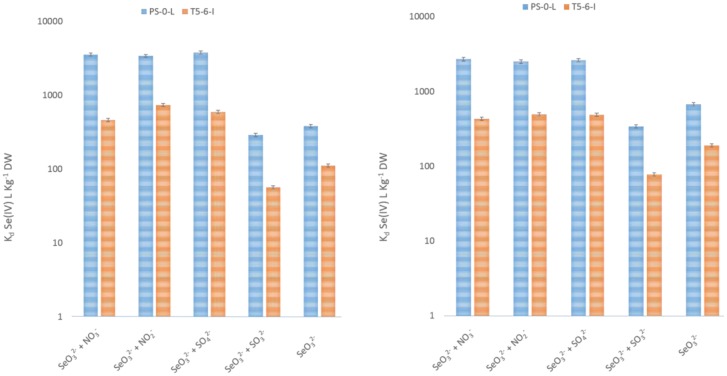
^75^Se(IV) uptake in *Pseudomonas* sp. PS-0-L and T5-6-I grown under aerobic conditions in the dark in 1% Tryptone in the presence of 6 mM NO_3_^−^, NO_2_^−^, SO_4_^2−^, SO_3_^2−^ and/or SeO_3_^2−^ at 20 °C (A) and 4 °C (B). Incubation time 7 days. The error indicated is a standard deviation of three parallel determinations.

Like other trace anions, Se(IV) may share transporters with major anions such as sulphate (SO_4_^2−^) and nitrate (NO_3_^−^). Therefore, we tested the roles of NO_3_^−^, nitrite (NO_2_^−^), SO_4_^2−^ and sulphite (SO_3_^2−^) in Se(IV) uptake and assumed that with up-regulation of NO_3_^−^/NO_2_^−^/SO_4_^2−^/SO_3_^2−^ the rate of Se(IV) uptake would increase in the presence of these anions. We found NO_3_^−^/NO_2_^−^/SO_4_^2−^ addition to enhance Se(IV) uptake in both *Pseudomonas* sp. strains, compared to the situation when only Se(IV) or Se(IV)O_3_^2−^ + SO_3_^2−^ were present (Figure 7). 3 mM NO_3_^−^, NO_2_^−^ and SO_4_^2−^ addition increased Se(IV) uptake on average 8-fold at 20 °C and 4-fold at 4 °C in both bacteria. 3 mM SO_3_^2−^ addition however decreased Se(IV) uptake on average by 30% at 20 °C and by 50% at 4 °C.

**Figure 8.**
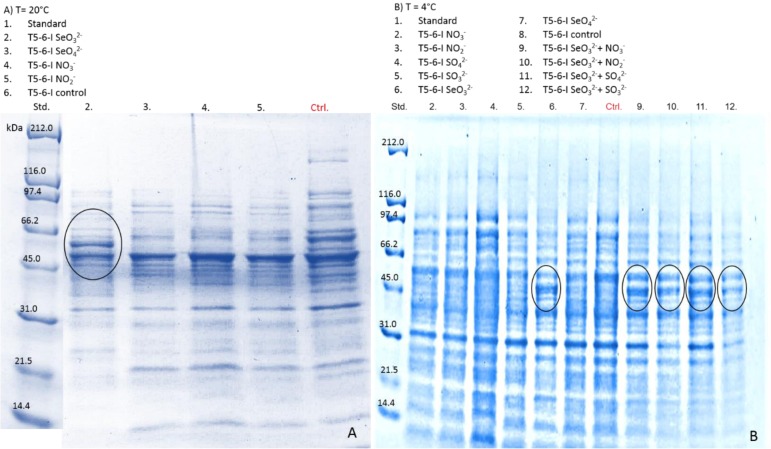
Total soluble protein fractions of *Pseudomonas* T5-6-I expressed in response to Se(IV) and other oxyanion treatments at 20 °C (A) and 4 °C (B). Black circles show changes in the protein expression and newly expressed proteins in the cells grown in the presence of Se(IV)O_3_^2−^. Equal amounts of protein (ca. 30 µg) were loaded onto each well.

The stress responses generated by Se(IV) addition were investigated to identify potentially important proteins involved in Se(IV) metabolism. In addition, the effect of NO_3_^−^/NO_2_^−^/SO_4_^2−^/SO_3_^2−^ and Se(VI) exposure as well as change in temperature from 20 °C to 4 °C on gene expression was examined by isolating the soluble proteins and inclusion body fractions from the cells grown with NO_3_^−^/NO_2_^−^/SO_4_^2−^/SO_3_^2−^ and Se(IV)/Se(VI) either at 20 °C or 4 °C for 24 h or 7 days. Moderations in the soluble protein patterns of *Pseudomonas* sp. T5-6-I strain were observable after 7 days incubation in the cells with visible red Se^0^ formations (Figure 4). Moderations depended in addition to differences in oxyanion content, also on incubation temperature. At 20 °C an approximate 65 kDa protein was expressed in the *Pseudomonas* sp. T5-6-I cells grown with Se(IV) (Figure 8 A, line 2). On the contrary, in the cells grown with Se(VI), NO_3_^−^ or NO_2_^−^, expression of this protein was repressed (Figure 8A, lines 3–5). In the cells of T5-6-I incubated at 4 °C, additional soluble protein of the size 43–45 kDa, was expressed in cells incubated with only Se(IV) or with Se(IV) + NO_3_^−^/NO_2_^−^/SO_4_^2−^/SO_3_^2−^ (Figure 8B, lines 6, 9–12). In the absence of Se(IV) this protein was not expressed (Figure 8B, lines 2–5, 7–8).

The *Pseudomonas* sp. PS-0-L strain displayed somewhat divergent protein profiles from those observed for the T5-6-I strain and as cells were exposed to Se(IV) at 4 °C or 20 °C, no clear alterations in the protein profiles were observed compared to the situation when Se(IV) was absent from the growth media, but other oxyanions, NO_3_^−^, NO_3_^2−^, SO_4_^2−^, SO_3_^2−^ or Se(VI) were added (data not shown). In this strain multiple proteins of the size 40–60 kDa were expressed at both 4 °C and 20 °C (data not shown), which resembled the protein patterns seen in T5-6-I strain after growing at 4 °C in the presence of Se(IV).

## 4. Discussion

There are various hypotheses about the uptake mechanisms of Se(IV) of different bacteria, including transport via a sulphate ABC transporter complex and reduced selenium (Se^0^) formed in the periplasmic compartment as well as the use of various enzymes, such as sulphite reductase and OYE enzyme [32]. It is however likely that alternative still unidentified carriers also exist, as for example the repression of sulphate ABC transporter does not completely inhibit Se(IV) uptake [32]. The reduction of Se(VI) or Se(IV) into selenide (Se(II)^2−^) is employed in some essential metabolic processes, such as the synthesis of selenocysteine, an important part of certain enzymes [32],[33]. As a microbial process analogous to denitrification, Se(VI) or Se(IV) can be used as first step electron acceptor in anaerobic respiration [16]. This is however observed only for a limited number of bacterial species (e.g. *Thauera selenatis* [34]). In addition, some anaerobic and phototrophic aerobic bacteria have previously been shown to be able to use selenium oxyanions as terminal electron acceptors [11],[18]–[23]. Se(VI) or Se(IV) reduction into insoluble, nontoxic elemental selenium (Se^0^) can in addition be utilized by various bacterial species to overcome the toxic effects of these selenium oxyanions.

We have previously shown that the two *Pseudomonas* strains T5-6-I and PS-0-L were able to remove ^75^Se(IV) from 1% Tryptone and 1% Yeast extract broths depending on incubation time and temperature [29]. In the present study, we have tested these strains further in order to identify which parameters stimulate or inhibit this removal process and resolve the fate of the selenium in this process.

Glucose stimulated Se(IV) accumulation in the tested bacterial strains and the amount of accumulated selenium depended on temperature (Figure 2). These findings indicate active transport of Se(IV) by the bacterial cells. However, as both bacterial strains were able to accumulate Se(IV) also in the absence of glucose and as they showed no glucose utilization ability in RapID^®^-tests [29], metabolism independent mechanisms (sorption on cell walls, diffusion inside the cell) for Se(IV) uptake should also be considered. Energy-independent mechanisms would, however, be affected by the pH of the solution, as pH affects the protonation of the cell wall as well as the selenium species. The effect of pH on Se(IV) uptake was therefore tested and we found that the varying pH did not significantly influence the removal Se(IV) from the solution by the *Pseudomonas* sp. PS-0-L strain. Nevertheless, it is unlikely that Se(IV) uptake would proceed via adsorption or simple diffusion, since the negatively charged cell membrane would repel anionic selenium species (SeO_3_^2−^, HSeO_3_^−^) in the pH of our samples (pKa values for H_2_SeO_3_ pK_a1_ = 2.5, pK_a2_ = 7.3).

Active uptake mechanisms are affected by various inhibitors. We used the sulphur-containing amino acid L-cysteine to inhibit Se(IV) uptake in the two *Pseudomonas* sp. strains and observed uptake of reduced Se(IV) even after L-cysteine addition by both strains. This observation is similar to the results reported for yeast *Saccharomyces cerevisiae*, in which the addition of 100 µM cysteine inhibited active transport of Se(IV) [35]. Furthermore, it has also been reported that cysteine addition inhibited growth in *E. coli* through interaction with membrane bound respiratory enzymes or through mechanisms interfering with leucine, isoleucine, threonine and valine biosynthesis [36] and aerobic (but not anaerobic) growth in *Rhodobacter sphaeroides* [11]. This differs from our observation, according to which L-cysteine inhibited only Se(IV) uptake, but no inhibition of growth was observed in the aerobic *Pseudomonas* PS-0-L and T5-6-I cells. This indicates that Se(IV) uptake in these bacteria may be regulated by cellular products formed in the sulphur metabolism. Therefore, we examined Se(IV) uptake also in the presence of SO_3_^2−^ or SO_4_^2−^ as well as two other oxyanionic macronutrients, NO_3_^−^ and NO_2_^−^, to determine whether these anions interfered with or enhanced uptake. For example, in *T. selenatis*, the reduction of Se(IV) has been suggested to be catalysed by a periplasmic nitrite reductase and in *Clostridium pasteurianum* by inducible sulphite reductases [37],[38]. We investigated the hypothetical involvement of NO_3_^−^, NO_2_^−^ and SO_4_^2−^ and SO_3_^2−^ reductases in the reduction of Se(IV) by analyzing the effects of NO_3_^−^, NO_2_^−^ and SO_4_^2−^ and SO_3_^2−^ amendments on the uptake of ^75^Se(IV) as well as on protein expression. In addition, the differences in reduction pathways between Se(IV) and Se(VI) were investigated by comparing the protein profiles and selenium appearance in TEM images in bacteria grown either in Se(IV) or Se(VI) containing solutions. We observed an enhanced uptake of Se(IV) induced by all other tested oxyanions with the exception of SO_3_^2−^, which inhibited the uptake. This is in line with earlier observations of Hudman and Glenn [39], who reported inhibition of selenite transport in *Selenomonas ruminantium* in the presence of sulphite, but not by nitrate or sulphate. However, in contrast to the *Pseudomonas* strains tested in our study, selenite transport in *S. ruminantium* was inhibited by nitrite. Nevertheless, it has been reported that sulphite stimulates selenite transport in *Salmonella typhimurium* and *E. coli* [40]. It therefore appears that distinct Se(IV) uptake, reduction and incorporation mechanisms are found among different bacterial genera. In our study the enhanced Se(IV) uptake in the presence of SO_4_^2−^, NO_3_^−^ and NO_2_^−^ and inhibitive effect of SO_3_^2−^ indicate the existence of two different transport pathways for Se(IV) in these two *Pseudomonas* sp. strains; (1) a low affinity transport system up-regulated by SO_4_^2−^, NO_3_^−^ and NO_2_^−^, which does not discriminate between Se(IV) and SO_4_^2−^, NO_3_^−^ or NO_2_^−^, and, (2) supported by the fact that Se(IV) uptake takes place also during SO_4_^2−^, NO_3_^−^ and NO_2_^−^ starvation, another Se(IV) regulated/induced uptake pathway. These observations were also supported by the findings obtained from the protein expression experiments, using the same anionic competitors.

The oxidative stress caused by the addition of Se(IV) [13] can be overcome by an increase in the synthesis of proteins involved in the cellular antioxidant defense. In *R. sphaeroides* the formation of reactive oxygen species has been reported to be detoxified by inducing the expression of an iron-containing superoxide dismutase -enzyme (SOD) [11]. In our study, an additional ∼43–45 kDa protein was expressed in the *Pseudomonas* T5-6-I strain, after Se(IV) treatment. In *E.coli* and *Saccharomyces cerevisiae* Se(VI)O has been reported to be transported into the cell through a SO_4_^2−^ permease system, which is in agreement with the similarities in the chemical properties of S(VI)O_4_^2−^ and Se(VI)O_4_^2−^ [41]. However, Se(IV) transport seems to be carried out by an alternative transport system and evidence of a ca. 43 kDa polypeptide involved in the transport has been reported in *E. coli* [42], which corresponds to the ∼43–45 kDa protein observed in our study. Correspondingly, Se(VI) and Se(IV) transport has been concluded to occur through non-identical transport pathways also in various other bacterial species, like *R. sphaeroides* [11],[43], *Clostridium pasteurianum* [44] and *Salmonella enterica* [40].

Differences in ^75^Se(IV) uptake after Se(IV) and Se(VI)/SO_4_^2−^/SO_3_^2−^/NO_3_^−^/NO_2_^−^ treatments under reduced temperature were observed in both *Pseudomonas* sp. strains examined in our study. In addition, changes in the protein expression after Se(IV) treatment was seen in *Pseudomonas* sp. T5-6-I. This indicates, that Se(IV) uptake by these strains may in addition to the oxidative stress response, be connected to the cold shock response and adaptation to low temperature. Earlier, Bebien et al. [11] reported that the addition of Se(IV) under dark aerobic conditions induced an important increase in the expression of heat shock proteins in *R. sphaeroides*. Heat shock proteins are essential components of the cellular protection against general stress and the induction of heat shock proteins is common under several stress factors like UV irradiation and heat stimuli [45],[46]. Similarly, cold shock proteins (Csps) are induced as a response to rapid temperature downshift (cold shock) [47]. In addition, some Csps are non-cold inducible and they have been reported to be involved in several cellular processes to support growth and stress adaptation responses [47]. These proteins have been reported to be involved in the tolerance to osmotic, oxidative, starvation, pH and ethanol stress as well as to host cell invasion [47]. Based on the temperature effect related to the Se(IV) uptake and induced proteins, found in our study, it is possible that same proteins in these bacteria are involved both on temperature adaptation and adaptation to various stress factors.

In addition, we observed *Pseudomonas* sp. T5-6-I and PS-0-L cells to efficiently reduce Se(IV) into metallic selenium under dark, aerobic conditions. This is in line with the earlier reported results obtained for *Rhodobacter sphaeroides* (a nitrogen fixing alphaproteobacterium) under aerobic, dark conditions [11],[48]. Both *Pseudomonas* sp. T5-6-I and PS-0-L cells accumulated metallic selenium in the cytoplasmic compartment after Se(IV) treatment, which implicates an intracellular reduction pathway for Se(IV) after transport through cell membrane. This is similar to earlier observations reported for *R. sphaeroides*, which was shown to accumulate intracellular metallic selenium [11]. In contrast, for example *Rhodospirillum rubrum* seems to excrete the selenium granules across the plasma membrane and the cell wall after completion of the selenite reduction [20]. The significant differences in the Se(IV) and Se(VI) reduction ability and protein expression after Se(IV) and Se(VI) treatment support the observations obtained from the uptake experiments with ^75^Se(IV) and competing macro-anions indicating two distinct transport and reduction systems for these oxyanions in the two *Pseudomonas* sp. strains investigated in our study.

## 5. Conclusions

Our results show conclusive differences on the selenium oxyanion, Se(IV) and Se(VI), reduction in these two *Pseudomonas* sp. strains, indicating distinctive uptake pathways for Se(IV) and Se(VI). In addition, demonstrated similarities (i.e. effect of Se(IV) concentration, glucose and cysteine as well as regulation affected by anionic macronutrients) in the uptake and reduction pathways between the two *Pseudomonas* sp. strains were found. These indicate uniform, conserved uptake mechanisms between these two strains of this genus. However, as significant differences in the protein expression patterns of the two *Pseudomonas* sp. strains after Se(IV) and temperature treatments were also observed, parts of the pathway seem to differ between the different strains. Se(IV) amendment induced several changes in the protein profiles of *Pseudomonas* sp. T5-6-I strain and newly expressed proteins were observed in the ∼65 kDa and ∼45 kDa regions. In addition, incubation temperature affected the expression of proteins in this bacteria and it is possible that these proteins are connected to the various stress factors, such as the adaptation of bacteria on low temperatures, typically found in the temperate boreal environments from where this *Pseudomonas* sp. strain was previously isolated, and oxidative stress.

Accumulation and transport in these *Pseudomonas* sp. strains is likely metabolism-dependent and two different transport mechanisms for Se(IV) uptake are suggested: (1) A low affinity transport system up-regulated by NO_3_^−^, NO_2_^−^ or SO_4_^2−^, and (2) a Se(IV) regulated transport system, since Se(IV) uptake also took place during sulphur and nitrogen starvation. Se(IV) was efficiently reduced in the cytoplasm following transport across the cell membrane, but the redox state of Se(VI) was not significantly affected. Based on the observed differences in reduction ability and relative toxicities of Se(IV)/Se(VI) it is presumed that these *Pseudomonas* sp. strains use Se(IV) reduction as a detoxification mechanisms.
